# Markers of Pluripotency in Human Amniotic Epithelial Cells and Their Differentiation to Progenitor of Cortical Neurons

**DOI:** 10.1371/journal.pone.0146082

**Published:** 2015-12-31

**Authors:** Irma Lydia García-Castro, Guadalupe García-López, Daniela Ávila-González, Héctor Flores-Herrera, Anayansi Molina-Hernández, Wendy Portillo, Eva Ramón-Gallegos, Néstor Fabián Díaz

**Affiliations:** 1 Laboratorio de Citopatología Ambiental, Escuela Nacional de Ciencias Biológicas, Instituto Politécnico Nacional, Campus Zacatenco, Unidad Profesional “Adolfo López Mateos”, México D.F., México; 2 Departamento de Biología Celular, Instituto Nacional de Perinatología, Montes Urales 800, Col. Lomas Virreyes, CP 11000, México D.F., México; 3 Departamento de Inmuno-Bioquímica, Instituto Nacional de Perinatología, Montes Urales 800, Col. Lomas Virreyes, CP 11000, México D.F., México; 4 Departamento de Neurobiología Conductal y Cognitiva, Instituto de Neurobiología, UNAM, Querétaro, México; Friedrich-Loeffler-Institute, GERMANY

## Abstract

Human pluripotent stem cells (hPSC) have promise for regenerative medicine due to their auto-renovation and differentiation capacities. Nevertheless, there are several ethical and methodological issues about these cells that have not been resolved. Human amniotic epithelial cells (hAEC) have been proposed as source of pluripotent stem cells. Several groups have studied hAEC but have reported inconsistencies about their pluripotency properties. The aim of the present study was the *in vitro* characterization of hAEC collected from a Mexican population in order to identify transcription factors involved in the pluripotency circuitry and to determine their epigenetic state. Finally, we evaluated if these cells differentiate to cortical progenitors. We analyzed qualitatively and quantitatively the expression of the transcription factors of pluripotency (OCT4, SOX2, NANOG, KLF4 and REX1) by RT-PCR and RT-qPCR in hAEC. Also, we determined the presence of OCT4, SOX2, NANOG, SSEA3, SSEA4, TRA-1-60, E-cadherin, KLF4, TFE3 as well as the proliferation and epigenetic state by immunocytochemistry of the cells. Finally, hAEC were differentiated towards cortical progenitors using a protocol of two stages. Here we show that hAEC, obtained from a Mexican population and cultured *in vitro* (P0-P3), maintained the expression of several markers strongly involved in pluripotency maintenance (OCT4, SOX2, NANOG, TFE3, KLF4, SSEA3, SSEA4, TRA-1-60 and E-cadherin). Finally, when hAEC were treated with growth factors and small molecules, they expressed markers characteristic of cortical progenitors (TBR2, OTX2, NeuN and β-III-tubulin). Our results demonstrated that hAEC express naïve pluripotent markers (KLF4, REX1 and TFE3) as well as the cortical neuron phenotype after differentiation. This highlights the need for further investigation of hAEC as a possible source of hPSC.

## Introduction

Human pluripotent stem cells (hPSC) have the capacity of self-renewal and differentiate into derivates of the three embryonic layers. These attributes of hPSC make them suitable candidates for possible application in regenerative medicine, as well as, for use in studies of developmental biology. The human embryonic stem cells (hESC) derived from the inner cell mass of the embryo and the induced pluripotent stem cells (iPSC) obtained from reprogramming a somatic cell are the most studied types of these cells. However, there are still several concerns with their possible use in regenerative medicine, including tumorigenicity, immunocompatibility between donor and receptor, ethical issues, and costly reprogramming, among others [1]. For these reasons alternative sources of hPSC have been proposed. In particular, human amniotic epithelial cells (hAEC), obtained from fetal membranes, have been reported to be positive for hPSC markers such as: Octamer-binding protein 4 (OCT4), SRY-related HMG-box gene 2 (SOX2), NANOG, Stage Specific Embryonic Antigens 3 and 4 (SSEA3, SSEA4), Tumor Rejection Antigen 1–60 (TRA1-60), Reduced Expression 1 (REX1) and E-cadherin [2–4]. hAEC are also able to differentiate into three embryonic layers [3–6]. However, precisely defining the identity and differentiation potential of stem cells from diverse sources has proven to be difficult, given the different sets of specific markers, protocols used and lack of information about side-by-side characterization of these cells. The findings of previous studies about hAEC are inconsistent with regard to their differentiation capacity as well as the presence or absence of stem cell markers [3, 7, 8]. Furthermore, the operational definition of pluripotency is no longer clear due to the revelation that there are a number of distinct cellular states that display these features, i.e., naïve and primed pluripotency involving different transcription factors and epigenetic states [9, 10]. These findings were not considered in the previous studies involving hAEC. The aim of the present study was the *in vitro* characterization of hAEC collected from a Mexican population in order to identify transcription factors involved in the pluripotency circuitry and to determine their epigenetic state. Finally, we evaluated if these cells differentiate to cortical progenitors.

## Materials and Methods

All experiments were carried out in accordance with the “Reglamento de la Ley General de Salud en Materia de Investigacion para la Salud” of the Mexican Health Ministry that follows NIH guidelines and approved by Ethics Committee of the National Institute of Perinatology. Fetal membranes were collected after elective cesarean delivery. Written patient consent and ethical approval were obtained before tissue collection, in accordance with the Ethics Committee of the National Institute of Perinatology guidelines, protocol 212250–21041. Women with uncomplicated, full term (37–40 weeks) pregnancies who did not experience activation of labor or premature rupture of membranes were included in this study. Also, a study of ancestry for at least three generations of ancestor born in Mexico was made to the patients to define Mexican population. No evidence of microbiological signs of chorioamnionitis or lower genital tract infection was found in the fetal membranes.

### Cell isolation and culture of hAEC

Fetal membranes were transported to the laboratory in sterile Hank’s balanced salt solution (HBSS, Life Technologies, GIBCO Grand Island NY, USA). The amnion layer was mechanically peeled off of the chorion and washed several times with HBSS to remove blood and clots. To obtain the hAEC we followed the protocol reported by Miki et al., with slight modifications [11]. Briefly, the amnion was incubated at 37°C with 0.05% trypsin EDTA (Life Technologies) for 10 min, followed by two, 40-min incubations with trypsin during which the conical centrifuge tube was shaken manually every 10 min. The enzyme was inactivated with fetal bovine serum (FBS, Life Technologies) in Dulbecco’s modified Eagle medium (DMEM, Life Technologies), and the cells were centrifuged for 10 min at 200 x g at 4°C. The pellet was resuspend in DMEM and filtered through a 100-μm cell strainer (Falcon Becton Dickinson, Franklin Lakes, NJ, USA). Finally, we counted the cells, and their viability was determined by trypan blue exclusion.

The cells were plated at a density of 3x10^4^ cells/cm^2^ on 100-mm culture plates (Corning, Corning, NY, USA) in DMEM supplemented with 10% FBS, 2 mM L-glutamine, 1% antibiotic-antimycotic, 1% sodium pyruvate, 1% nonessential amino acids and 55 μM β-mercaptoethanol (all from Life Technologies). hAEC were cultured at 37°C in normoxia conditions (5% CO2, 95% air) in a humidified incubator. The medium was changed every 3 days and 10 ng/ml Epidermal Growth Factor (EGF; Peprotech, Rocky Hill, NJ, USA) was supplemented every day. Cultures were grown to near confluence, and three cell passages (P0, P1, P2, and P3) were performed using trypsin/EDTA. At beginning of each passage, we reseeded at a density of 3x10^4^ cells/cm^2^.

### Culture of hESC

The hESC lines, H1 (WB0194) and H9 (WB0143) were obtained from WiCell Research Institute, Inc. The cells were cultured and maintained by enzymatic passaging on mouse embryonic fibroblast (MEF) feeder layers according to standard WiCell protocols. The hESC were used as pluripotency marker control.

### Differentiation of hAEC towards cortical progenitors

We followed the protocol modified from Lippman et al. [12] that consists in proliferation and differentiation stages. At the first stage, P1 hAEC were incubated for 8 days in serum-free Insulin-Transferrin-Selenite medium (Life Technologies) supplemented with 1% antibiotic-antimycotic (basal medium), and six conditions were tested: 1) basal medium, 2) 10 ng/ml basic Fibroblast Growth Factor (bFGF; Peprotech), 3) bFGF plus 10 ng/ml of SB431542 (Stemgent, Cambridge, MA, USA), 4) bFGF plus 10 ng/ml Noggin (Sigma-Aldrich, St. Louis, MO, USA), 5) bFGF plus 10 ng/ml EGF and 6) bFGF, EGF, SB431542 and Noggin. At the differentiation stage, the cells were cultured for 6 days in serum-free N2 medium (Life Technologies) in which all the factors were withdrawn.

To evaluate differentiation to the neural linage, the expression of Nestin was detected by immunofluorescence at the end of the proliferation stage. We tested for the progenitor of cortical neuron markers Otx2, β-III-tubulin, TBR2 and NeuN at the end of the differentiation stage.

### Reverse transcriptase-polymerase chain reaction (RT-PCR) and Quantitative PCR (RT-qPCR)

The RT-PCR and RT-qPCR were carried out following our previously reported protocols [13, 14]. Briefly, total RNA was isolated from cultured cells using TRIzol (Life Technologies) and was reverse transcribed and amplified using a one-step RT-PCR system (Promega A1280, Madison, WI, USA) and the following forward (F) and reverse (R) primers (Integrated DNA Technologies IDT, Coralville, IA, USA). The primer sequences, temperature of annealing and expected size of the products (bp) were: *OCT4* F:5´-GAG GAG TCC CAG GAC ATG AA-3´, R:5´-GTG GTC TGG CTG AAC ACC TT-3´, TM = 56.7°C, 151 bp; *SOX2* F:5´-GCC GAG TGG AAA CTT TTG TC-3´, R:5´-GTT CAT GTG CGC GTA ACT GT-3´, TM = 55.25°C, 264 bp; *NANOG* F:5´-CAG CTG TGT GTA CTC AAT GAT AGA TTT-3´, R:5´-CAA CTG GCC GAA GAA TAG CAA TGG TGT-3´, TM = 54.7°C, 286 bp; *REX1* F:5´-GCG TAC GCA AAT TAA AGT CCA GA-3´, R: 5´-CAG CAT CCT AAA CAG CTC GCA GAA T-3´, TM = 57.5°C, 306 bp; *KLF4* F:5´-GAT GGG GTC TGT GAC TGG AT-3´, R:5´-CCC CCA ACT CAC GGA TAT AA-3´, TM = 55.1°C, 134 bp. GAPDH F:5´-ATC ACC ATC TTC CAG GAG CG-3´, R:5´-CCT GCT TCA CCA CCA CCT TCT TG-3´, TM = 56.75°C, 229 bp.

Cycling parameters were as follows: denaturalization at 95°C for 2 min, annealing at specific temperatures for 1 min and elongation at 72°C for 1 min. The number of cycles was 35 for all the genes. Final extension at 72°C was for 5 min and was terminated by rapid cooling to 4°C. PCR products were analyzed in a 2% agarose gel, and their sizes were determined by comparison with molecular weight standards after GelRed (Biotium, Hayward, CA, USA) stain; the results were captured with a gel documentation system (DNR Bio-Imaging System, Jerusalem, Israel) using GelCapture Acquisition software (DNR Bio-Imaging Systems). As negative control for PCR amplification, reactions with RNA in the absence of reverse transcriptase as well human peripheral blood and mouse embryonic fibroblast cells were included.

In another series of experiments, RT-qPCR was performed using the same primer sequences. Total RNA was isolated using TRIzol (Life Technologies) and reverse transcribed into cDNA with oligo dT (Life Technologies). The qPCR-amplification parameters were the same as for end-point PCR reactions, but using KAPASYBR^®^ FAST Universal qPCR Master Mix (KAPA Biosystems, Boston, MA, USA). qPCR was carried out in a Rotor-Gene Q 7000 thermocycler using 800 ng of cDNA (Qiagen, Valencia, CA, USA). At the end of the run, melting curves were performed for each reaction to ensure the amplification of a single product. To validate the method, we performed dynamic range curves with 10 ng to 1000 ng of each gene, obtaining an average efficiency = 1.57 and R^2^ = 0.98. Reactions with no template were used as negative controls. We determined a Ct value = 0.2 and applied it for every qPCR experiment. Gene expression data were normalized to the GAPDH housekeeping gene as a reference using the 2^-ΔΔCT^ method. Reactions with total RNA were used as negative controls.

### Immunocytochemistry

Immunofluorescence experiments were carried out following our previously reported protocols [14, 15]. Briefly, cells were fixed with 4% paraformaldehyde and permeabilized using 0.3% Triton X-100 (Sigma-Aldrich) diluted in PBS. To block unspecific epitopes, cells were incubated with 5% bovine serum albumin (BSA, AMRESCO, Solon, OH, USA). Primary antibodies to evaluate pluripotency were: rabbit anti-OCT4 (1:100, Cat. ab19857, Abcam, San Francisco, CA, USA), rabbit anti-SOX2 (1:150, Cat. ab97959, Abcam), rabbit anti-NANOG (1:500, Cat. 500-P236, Peprotech), rabbit anti-Kruppel-Like Factor (KLF) 4 (1:200, Cat. sc-20691, Santa Cruz Biotechnology, Dallas, TX, USA), rabbit anti-transcription factor binding to immunoglobulin heavy constant mu (IGHM) enhancer 3 (TFE3) (1:100, Cat. ab97667, Abcam), rat anti-SSEA3 (1:50, Cat. ab16286, Abcam), mouse anti-SSEA4 (1:150, Cat. ab16287, Abcam), mouse anti-TRA-1-60 (1:150, Cat. ab16288, Abcam) and mouse anti-E-cadherin (1:50, Cat. 610181, BD Biosciences, San Jose, CA, USA). The epigenetic state was determined by the presence of trimethylation of the lysine 27 on histone 3 (rabbit anti-H3K27me3, 1:500, Cat. ABE44, Merck Millipore, Billerica, MA, USA) and trimethylation of the lysine 4 on histone 3 (mouse anti-H3K4me3, 1:1000, Cat. ab6000, Abcam). In all cases, double staining was performed with the antibody against Ki67 (1:100, Cat. sc-23900 and sc-15402, Santa Cruz). To determine the differentiation of the progenitor of cortical neurons, we used the following antibodies: mouse anti-Nestin (1:200, Cat. GTX30671, GeneTex, Irvine, CA, USA), mouse anti-NeuN (1:100, Cat. ab104224, Abcam), rabbit anti-TBR2 (1:200, Cat. ab23345, Abcam), rabbit anti-OTX2 (1:400, Cat. ab21990, Abcam) and mouse anti-β-III-tubulin (1:2000, Cat. sc-51670, Santa Cruz). Primary antibodies were incubated overnight at 4°C followed by incubation with appropriate fluorescently labeled secondary antibodies for 2 h at room temperature. Finally, cell nuclei were counterstained with 4',6-diamidino-2-phenylindole (DAPI). Negative controls consisted of cultures in which the primary antibody was omitted. These experiments did not produce any stain (data not shown). H1 cells were used as pluripotency controls.

### Cell counting

At the end of each passage (P0-P3), photomicrographs of the immunofluorescence were taken through an epi-fluorescence microscope (Olympus IX-81, Tokio, Japan) with a CCD camera (Hamamatsu, ORCA-Flash 2.8, Tokio, Japan). The number of cells that expressed the cellular markers of interest was evaluated in nine random fields taken at 200X, from three to five independent experiments. To establish co-expression of markers, merged images were generated. In these cultures, we quantified the total number of cells by counting the DAPI stained nuclei and the percentage of positive cells to each experiment according to following equation: % of positive cells = (number of single or double positive cells to a specific marker X 100) / total number of cell nuclei (DAPI positive).

### Statistical analysis

Data was not normally distributed and therefore was analyzed by Kruskal-Wallis test. *Post-hoc* comparisons were performed using the Mann-Whitney U test. Differences were considered statistically significant at p < 0.05. For the statistical analysis, the SigmaPlot 11.0 program was used (Systat Software Inc, San Jose, CA, USA).

## Results

### *In vitro* morphological characterization of hAEC

From each fetal membrane processed we were able to obtain an average of 80 x 10^6^ hAEC (n = 5). *In vitro*, these cells present a cobblestone epithelial morphology, and they proliferate until they form an adherent monolayer (S1A Fig). The culture became confluent at 7 days for P0 to P1 and for the subsequent passages, at 5 days. hESC morphology was in accordance with previous reports (S1B Fig) [16].

### Analysis of the presence of pluripotency transcription factors in hAEC

**Fig 1:**
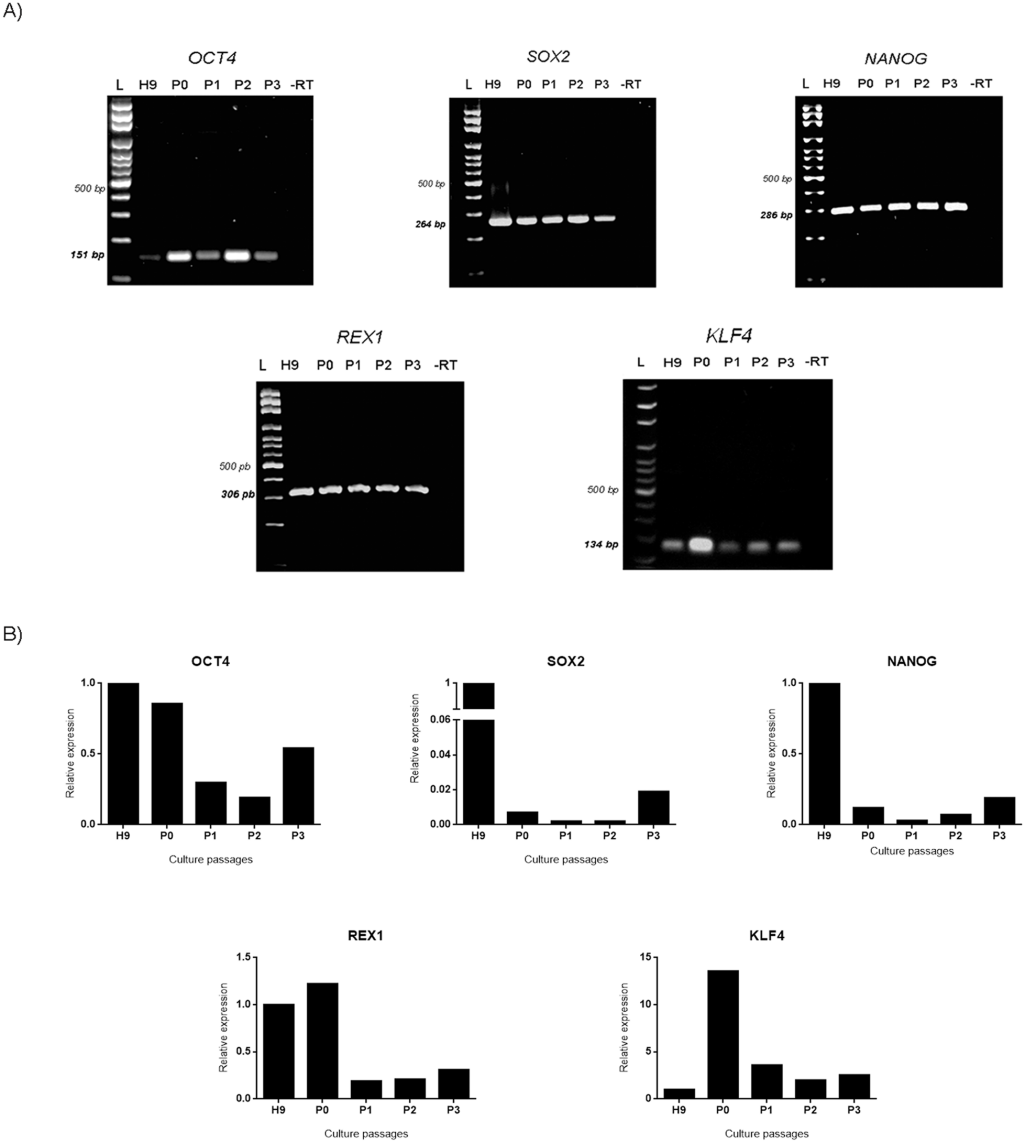
hAEC cultured *in vitro* express genes associated with pluripotency. (A) Representative images of the electrophoresis of RT-PCR products of mRNAs for transcription factors *OCT4* (151 bp), *SOX2* (264 bp), *NANOG* (286 bp), *REX1* (306 bp), *KLF4* (134 bp) of hAEC cultured *in vitro*. L = ladder, H9 = hESC line H9 (positive control), P = passage. As negative control (-RT), the reverse transcriptase enzyme was not added. (B) Relative expression levels obtained from RT-qPCR of the pluripotency genes (mean from 5 independent experiments).

Expression *of OCT4*, *SOX2*, *NANOG*, *REX1* and *KLF4* in H9 and hAEC from P0-P3 was analyzed qualitatively by RT-PCR. Our results showed that hAEC express all of these transcription factors in all passages, and the amplified products were of the predicted size (Fig 1A and S2 Fig). As expected, the hESC positive control also expressed these markers.

### Quantification of pluripotency factors in hAEC cultured *in vitro*

The relative expression for each gene was determined by RT-qPCR using the 2^-ΔΔCT^ method. The five genes analyzed were expressed by hAEC at all passages. However, no significant differences were found between passages (Fig 1B and S1 Table).

### Detection of the pluripotency markers in hAEC cultured *in vitro*

To validate our set of antibodies, we performed immunocytochemistry in hESC. All antibodies produce the staining reported in the literature [16] (S1C Fig).

**Fig 2:**
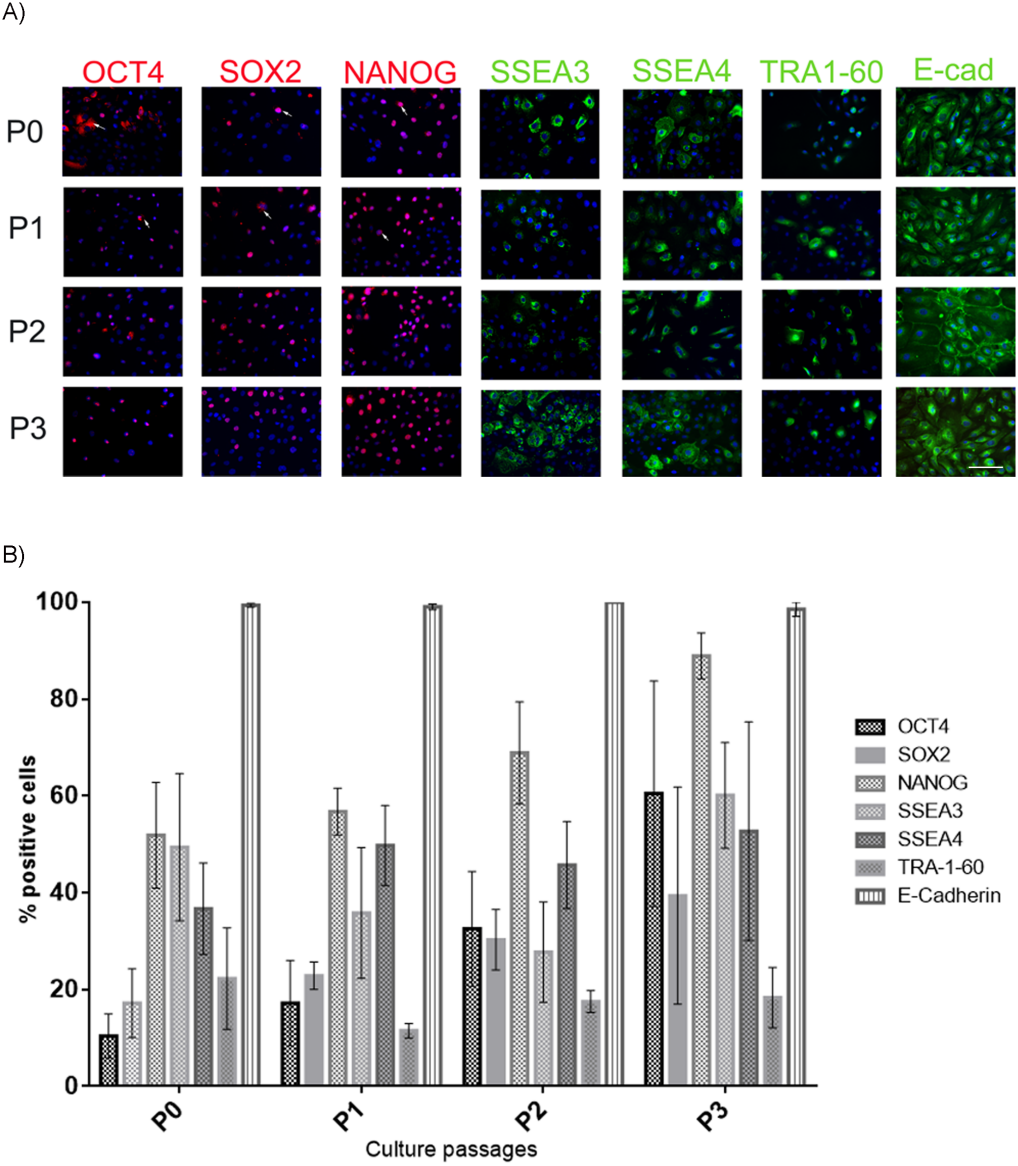
hAEC display the pluripotent stem cell markers. (A) Representative micrographs at 20X from hAEC at different passages (P0-P3) immunostained for OCT4 (red), SOX2 (red), NANOG (red), SSEA3 (green), SSEA4 (green), TRA-1-60 (green) and E-cadherin (green); the nuclei were stained with DAPI (blue). Arrow indicate that the marker was found in the cytoplasm. (B) Graph shows the percentage of hAEC positive for the different pluripotent markers. Results are expressed as percentages of means ± S.E.M. from 9 fields counted in duplicate from five independent experiments. Scale bar 50 μm.

The pluripotent markers OCT4, SOX2 and NANOG were detected in hAEC through passages P0-P3. Interestingly, in early passages, the markers were present in the cells' cytoplasmatic membrane and nucleus. However, in P3 the immunostaining was localized exclusively in the nucleus (Fig 2A). The percentage of hAEC that expressed OCT4, SOX2 and NANOG during the various passages did not change significantly (Fig 2B).

Surface antigens SSEA3, SSEA4, TRA-1-60 and E-cadherin were present in the hAEC cultures (Fig 2A). Similarly, quantification of the markers showed no significant differences among passages (Fig 2B).

### Proliferation of hAEC that present pluripotent markers *in vitro*

**Fig 3:**
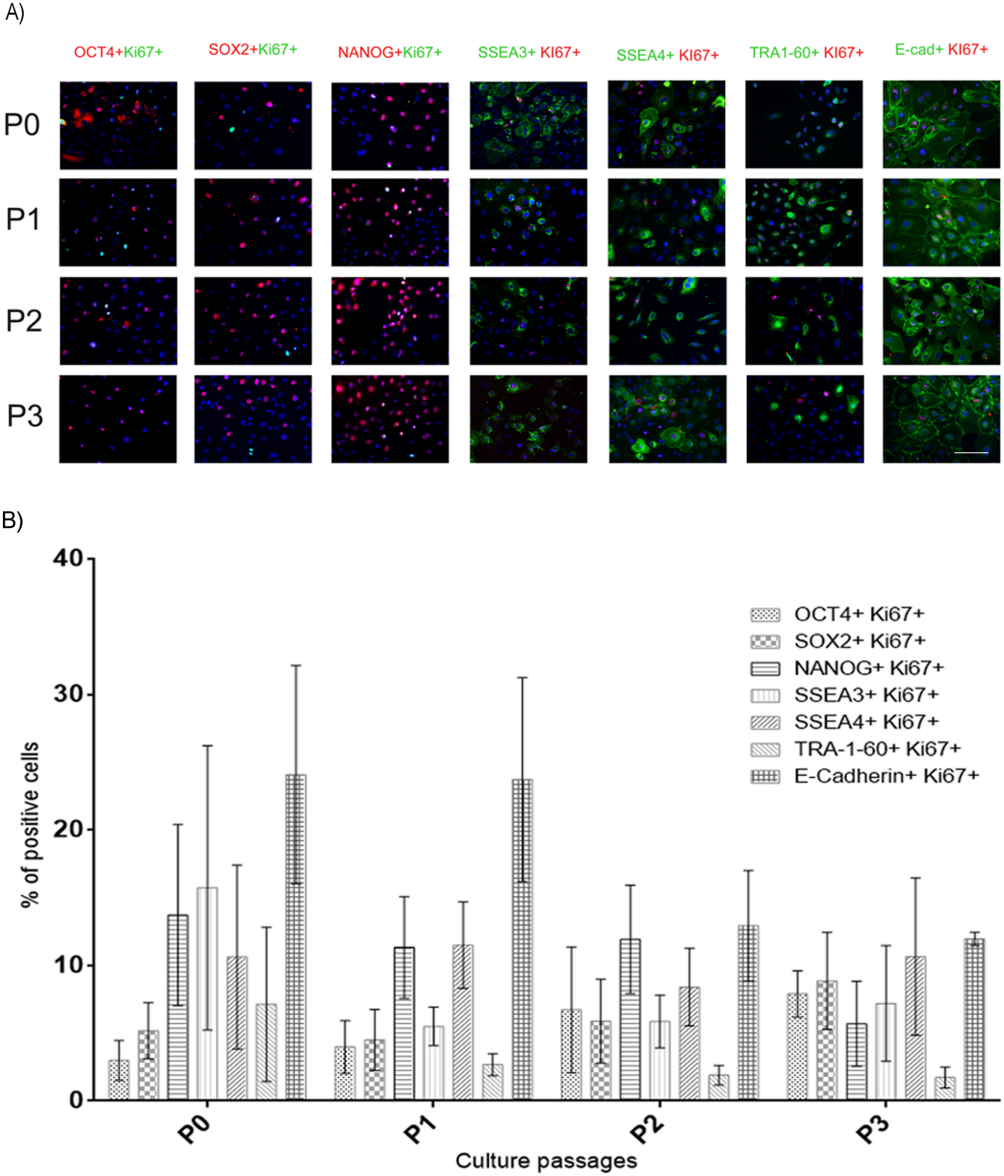
Proliferation of hAEC that display pluripotent markers *in vitro*. (A) Representative micrographs at 20X from hAEC at different passages (P0-P3) immunostained for OCT4 (red), SOX2 (red), NANOG (red), SSEA3 (green), SSEA4 (green), TRA-1-60 (green) and E-cadherin (green) and also for Ki67 (green or red); the nuclei were stained with DAPI (blue). (B) Graph shows the percentage of hAEC positive for one of the pluripotency factors and Ki67. Results are expressed as percentages of means ± S.E.M. from 9 fields counted in duplicate from five independent experiments. Scale bar 50 μm.

In order to determine if the OCT4-, SOX2-, NANOG-, SSEA3-, SSEA4-, TRA-1-60-positive hAEC were able to proliferate, we evaluated the number of these cells that co-expressed the protein Ki67 (nuclear protein present in proliferating cells) (Fig 3A). The percentage of cells expressing both Ki67 and pluripotency markers ranged from 2% to 24%. No significant differences were found between groups (Fig 3B).

### hAEC present naïve markers of pluripotency

**Fig 4:**
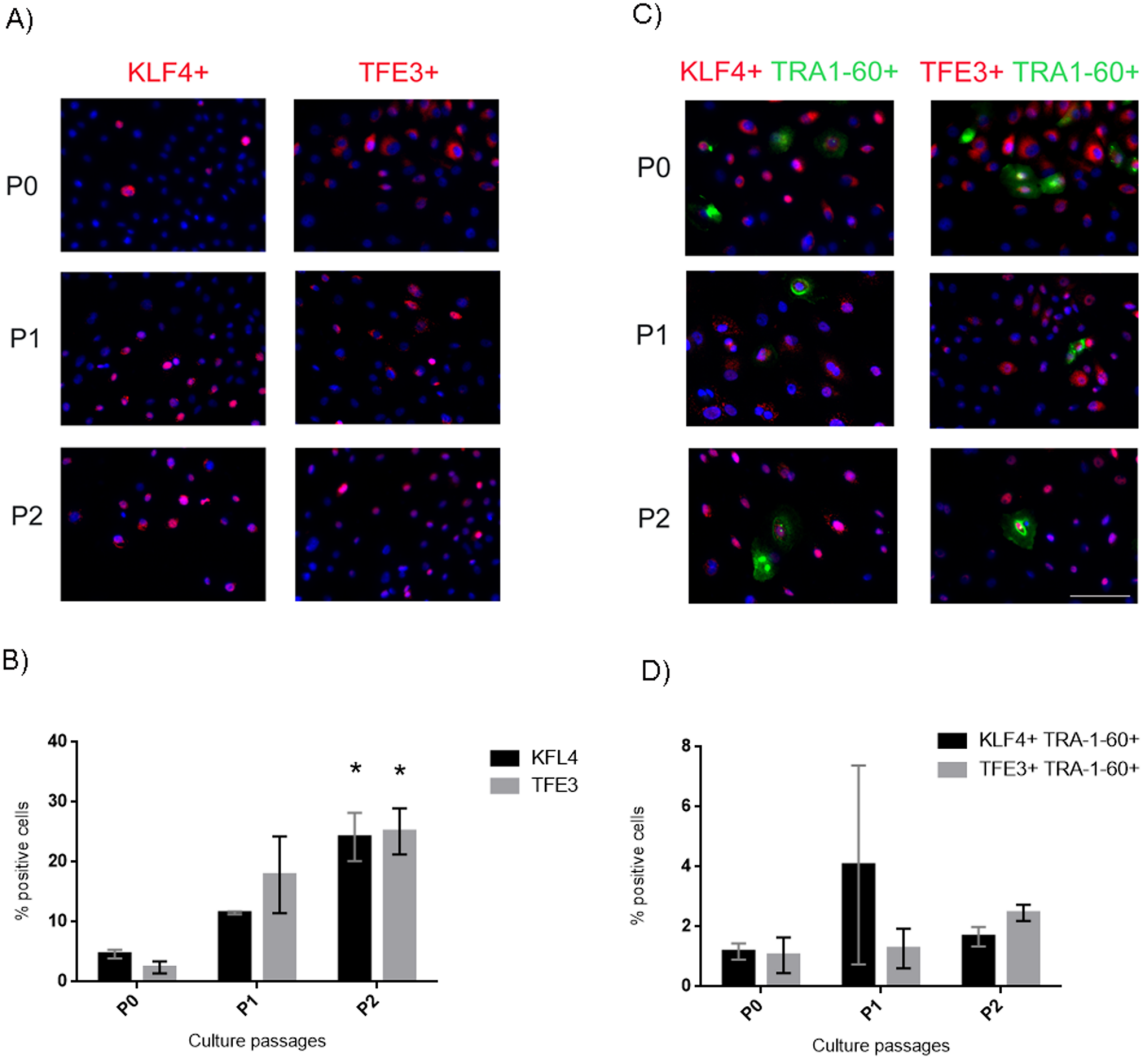
hAEC are positive for naïve pluripotent markers. (A) Representative micrographs at 20x from hAEC at P0-P2 immunostained for KLF4 (red) and TFE3 (red); the nuclei were stained with DAPI (blue). (B) Graph shows the percentage of hAEC that express naïve markers. (C) Representative micrographs at 20x from hAEC at P0-P2 immunostained for KLF4 (red) or TFE3 (red) and co-expressing TRA-1-60; the nuclei were stained with DAPI (blue). (D) Graph shows the percentage of cells immunostained for both TRA-1-60 and naïve markers. Results are expressed as percentages of means ± S.E.M. from 9 fields counted in duplicate from three independent experiments. Scale bar 50 μm. * p < 0.05 as compared with P0.

Once we determined the expression of pluripotent markers in our conditions, we tested for the presence of the naïve pluripotent markers, KLF4 and TFE3. Interestingly, these transcription factors were present in the hAEC from P0-P2, as shown by immunofluorescence (Fig 4A). Our results indicated a significant increase in the percentage of cells that express KLF4 and TFE3 in P2 in comparison with P0 (Fig 4B). We evaluated if KLF4- and TFE3-positive hAEC co-expressed TRA-1-60 (Fig 4C). The number of double-labeled cells showed that only ~ 2% of the cells were positive for both KLF4-TRA-1-60 and TFE3-TRA-1-60 at the end of P2 (Fig 4D).

### Epigenetic state of hAEC

**Fig 5:**
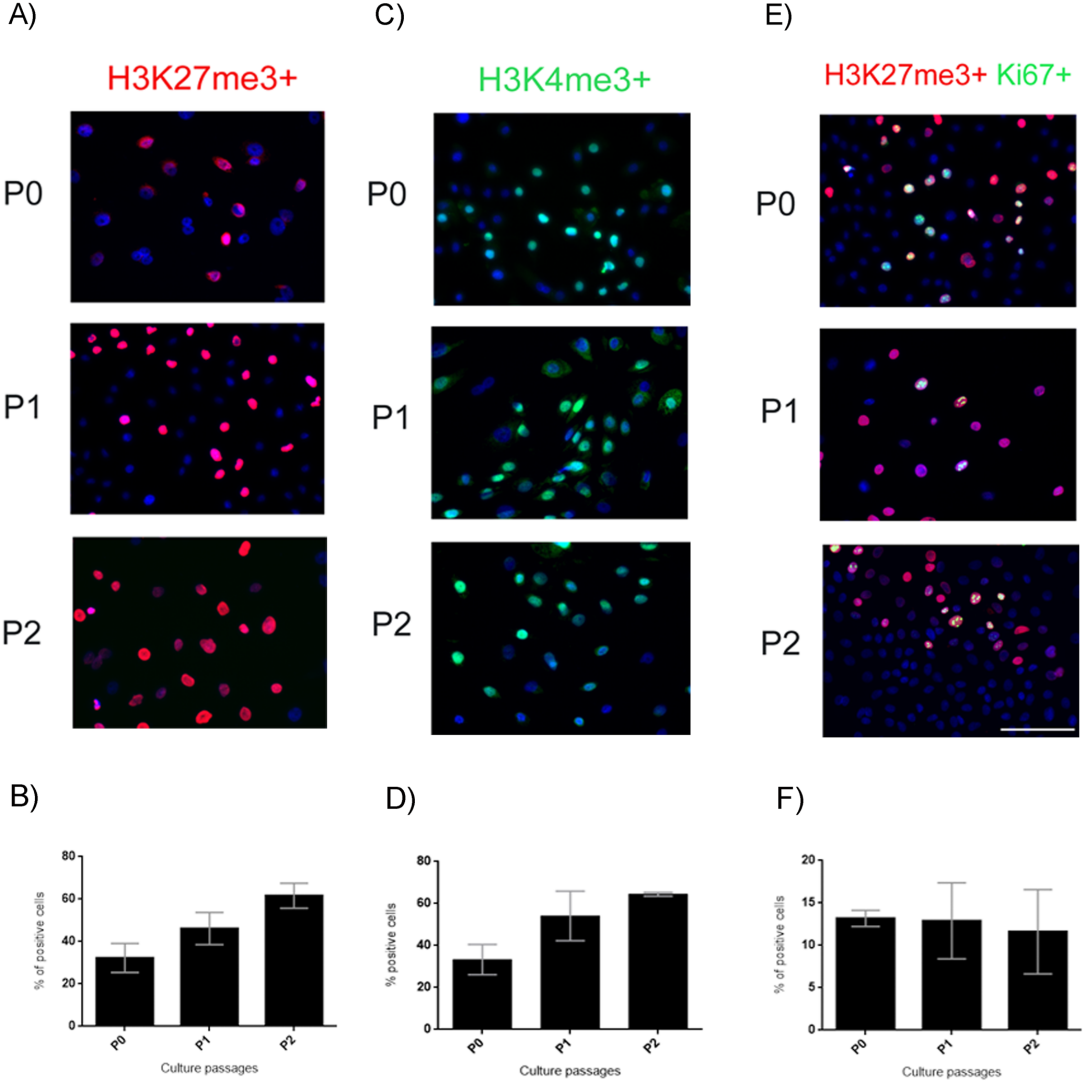
Epigenetic state of hAEC. (A) Representative micrographs at 20x from hAEC at P0-P2 immunostained for H3K27me3 (red); the nuclei were stained with DAPI (blue). (B) Graph showing the percentage of hAEC that present the H3K27me3 mark. (C) Representative micrographs at 20x from hAEC at P0-P2 immunostained for H3K24me3 (green); the nuclei were stained with DAPI (blue). (D) Graph showing the percentage of hAEC that present the H3K4me3 mark. (E) Representative micrographs at 20x from hAEC at P0-P2 immunostained for H3K27me3 (red) and Ki67 (green); the nuclei were stained with DAPI (blue). (F) Percentage of hAEC positive for both H3K27me3 and Ki67. Results are expressed as percentages of means ± S.E.M. from 9 fields counted in duplicate from three independent experiments. Scale bars, 50 μm.

To evaluate the methylation state of the hAEC we performed immunocytochemistry to detect H3K27me3 and H3K4me3 in our cultures (Fig 5A and 5C). H3K27me3 was present in 30% of the cells at P0 and increased to 60% at P2 (Fig 5B). H3K4me3 was present in 33% of the cells at P0 and increased to 64% at P2 (Fig 5D). The percentage of cells double-immunostained for H3K27me3 and Ki67 (Fig 5C) was maintained during the passages at ~12% (Fig 5D). Nonetheless, the increase of H3K24me3 and H3K3me3 from P0 to P2 is not significant.

### Differentiation of hAEC to cortical progenitors

**Fig 6:**
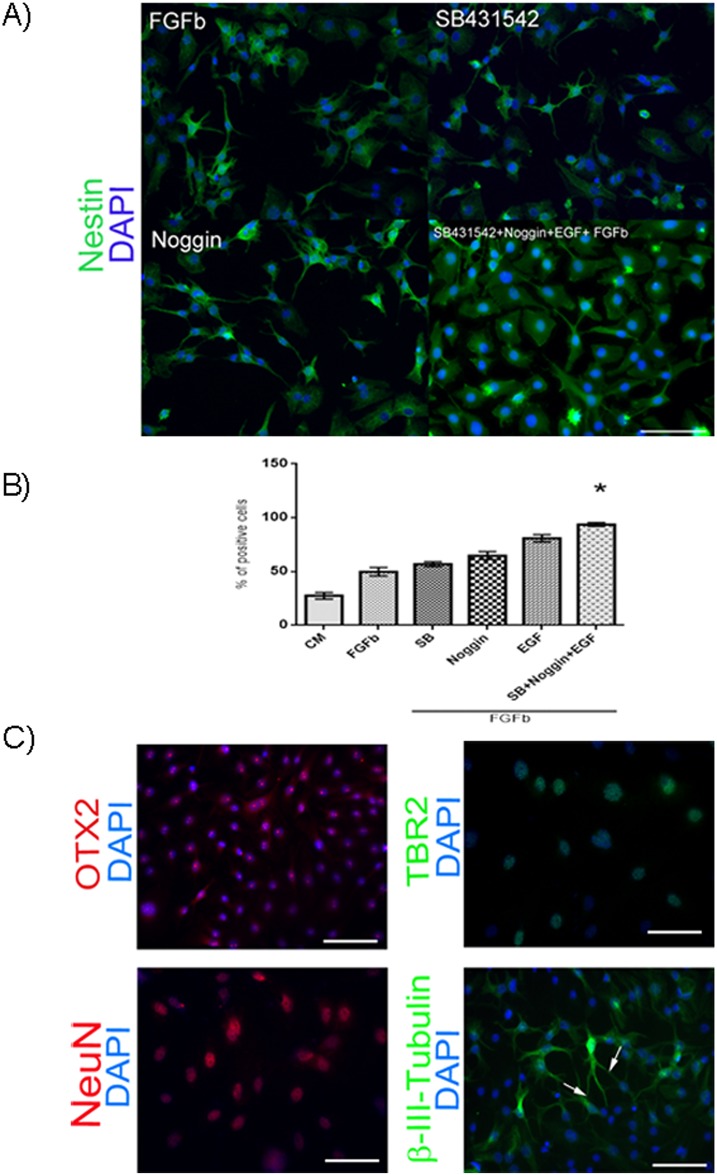
Differentiation of hAEC to progenitors of cortical neurons. (A) Representative micrographs at 20x from hAEC differentiated to Nestin-positive cells (green) after different treatments; the nuclei were stained with DAPI (blue). When the hAEC were treated with SB431542 + Noggin + EGF + bFGF the proportion of Nestin-positive cells increased. (B) Percentage of Nestin-positive cells obtained from hAEC during the proliferation stage with different treatments. Results are expressed as percentages of means ± S.E.M. from 9 fields counted in duplicate from three independent experiments. (C) Representative micrographs of hAEC treated with SB431542 + Noggin + EGF + bFGF. On the 14^th^ day, we found cells that were positive for OTX2 (red), TBR2 (green), NeuN (red), or β-III-tubulin (green); the nuclei were stained with DAPI (blue). Arrow indicate neurite prolongation. Scale bar 50 μm. * p < 0.05 as compared with basal medium (CM).

Finally, we differentiated hAEC to cortical progenitors. We tested 6 combinations of inhibitory molecules and growth factors in our cultures. At the first stage of the protocol, immunocytochemistry was performed to detect Nestin-positive cells after culturing in different conditions (Fig 6A). We found that the percentage of Nestin-positive cells increased significantly in medium containing bFGF+EGF+SB431542+Noggin versus control medium (Fig 6B). Cells from the bFGF+EGF+SB431542+Noggin condition were cultured for another 6 days without these molecules to induce cell differentiation. In these experiments we were able to identify cells expressing specific to progenitors of cortical neuron markers (OTX2, TBR2, β-III-tubulin, NeuN) by immunofluorescence (Fig 6C).

## Discussion

hAEC have been proposed as a reservoir of hPSC [2–4,7,17–19]. These cells have additional characteristics that make them a promising source for regenerative medicine. For example, the amnion is a medical waste after birth, and therefore, hAEC can be obtained in large quantities without ethical or legal issues. Furthermore, it is relatively easy to obtain these cells, as compared with protocols for pluripotent or multipotent stem cell derivation. Another important characteristic of hAEC is their secretion of bioactive molecules, including: angiogenic factors, immunosuppressive factors, and growth factors; these cells also have anti-inflammatory effects [8,18, 20].

### Pluripotent characteristics of hAEC

In our study, we evaluated the expression of *OCT4*, *SOX2*, *NANOG*, *KLF4* and *REX1* in hAEC through 4 passages (P0-P3), and we found that these cells express these markers consistently along all the passages. These findings are in accordance with previous reports [3, 7, 8, 21] and reinforce the possibility that cells isolated from human amniotic membranes have pluripotent characteristics. Interestingly, in our conditions, hAEC were able to expressed markers of the naïve state of pluripotency such as *KLF4*, *REX1* and *TFE3* [10, 22, 23]. To our knowledge, this is the first report that evaluated the expression of pluripotent naïve markers in hAEC. Also, we analyzed by RT-qPCR the relative expression of the three core pluripotency genes (*OCT4*, *NANOG and SOX2*) and naïve factors (*KLF4* and *REX1)*. However, no significant differences were found among the passages in the expression of these genes. Nevertheless, the relative expression of these genes showed a tendency to be higher at P0 than P1-P2 in all cases (Fig 1B). One possible explanation for the lower relative expression of these mRNAs after P0 could be adaptation of the cells to culture conditions.

As gene expression does not ensure the presence of the encoded protein, we analyzed for the proteins by immunofluorescence in hAEC at all 4 passages (P0-P3). We found that the hAEC were positive for several pluripotent marker proteins during the time analyzed (Fig 2). Previous reports have detected these markers by their mRNA or/and protein, but they were not quantified or were evaluated at only one point during culture. Miki et al. used qualitative PCR to measure the expression of OCT4, SOX2, FGF4 and REX1 in freshly isolated hAEC, and they only reported the levels of OCT4 and NANOG after 18 days of culture *in vitro*. Also, they quantified the number of cells positive for SSEA3 (9%), SSEA4 (44%), TRA-1-60 (10%) and TRA-1-81 (10%) by fluorescence-activated cell sorter; however, the number of cells positive for the transcription factor of pluripotency was not studied [3]. Ilancheran et al. performed qualitative PCR for the OCT4, SOX2, NANOG, DPPA3 and CFC1 mRNAs obtained from tissue and fresh hAEC; they also determined the presence of SSEA4, OCT4, SOX2 and GCTM2 by immunocytochemistry in hAEC cultured for 24 h but again, they did not report any quantification of these markers [7]. Zhou et al. evaluated by RT-PCR the expression of OCT4, SOX2, NANOG and KLF4 from hAEC at P1 and P2; they confirmed the presence of these proteins by immunocytochemistry only at P1, but there was no quantification [21]. Recently, Koike et al. detected the OCT4, SOX2, NANOG, KLF4, c-Myc and Lin28 mRNAs in hAEC by RT-qPCR and evaluated the presence of OCT4, SOX2, KLF4, c-Myc, SSEA3 and 4, TRA-1-60 and 1–81 proteins by immunohistochemistry in hAEC on the 7^th^ day of culture. They quantified the percentage of cells positive for SSEA3 (40%), SSEA4 (97%), TRA-1-60 (8%), and TRA-1-81 (5%) by flow cytometry [8]. In our study, we quantified the surface antigens and the transcription factors associated with pluripotency. We found that at P0, our cells were positive for OCT4, 10.4%; SOX2, 17.22%; NANOG, 51.9%; SSEA3, 49.4%; SSEA4, 36.7% and TRA-1-60, 22% (Fig 2B). These results are different from those previously reported [3,8]. It is important to mention that the primary antibodies as well as the methodology used in the other studies were different from those in the present study. For this reason, the specificity of our signal was confirmed by immunocytochemistry in hESC (S1 Fig). Also, we found that at the end of our cultures the percentages of positive cells were OCT4, 60.5%; SOX2, 39.41%; NANOG, 88.95%; SSEA3, 60.15%; SSEA4, 52.73%; TRA-1-60, 18.35%. No significant differences were found in the presence of these factors among the passages, but the results do show an increasing trend for the percentages of OCT4-, SOX2- and NANOG-positive cells at late passages. In contrast, TRA-1-60 expression was maintained with time in culture. Additionally, we evaluated our cultures for the presence of E-cadherin, another marker of hPSC that has not been previously measured in hAEC. Our hAEC populations were almost 100% positive for E-cadherin, which indicates that our cultures were not contaminated with other cellular types from the amnion or chorion. It has been reported that E-cadherin maintains pluripotent self-renewal properties and cell-to-cell contact [24], and the surface expression pattern of E-cadherin becomes more robust in naïve hESC colonies [9].

We also evaluated the presence of KLF4 and TFE3 in hAEC; these proteins have been detected in the naïve pluripotent state [10, 22, 23, 25–28]. We found a statistically significant increase in both markers at P2 in comparison with P0 (Fig 4B). Later, using double immunocytochemistry for TRA-1-60 and the naïve markers (KLF4 or TFE3) (Fig 4C and 4D), we observed that only 1–2% of the population was double positive for these markers at P0-P2. We measured TRA-1-60, because it has been reported that it is one of the first antigens expressed by induced pluripotent stem cells (iPSC) when they are reprogramming, and it is considered the bona fide iPSC stem cell marker [29, 30]. This percentage (1–2%) corresponds to the percentage of cells that co-express Ki67 and TRA-1-60 at end of P2 (Fig 3B), which suggests a population in a state of auto-renewal. Together, this evidence suggests the existence of a minor subpopulation of hAEC that has naïve pluripotent markers. Embryogenesis is inherently different between species, which is reflected by the difficulties in generating truly naïve hPSC *in vitro* [31]. For ethical reasons, information on human embryogenesis is lacking, and many assumptions are made based in the mouse model; considering this, hAEC could be a good model to study the naïve and primed pluripotent cells in humans. However, further studies are needed to determine the existence of naïve or primed populations in hAEC *in vitro* conditions.

Previous studies have reported that cultures obtained from the amnion are composed of a heterogeneous population [5, 8, 19]. Our results are consistent with and support the presence of different cell subpopulations derived from the human amniotic epithelial layer. The percentage of each pluripotent marker varies between passages and between biological samples; the absence of significant differences in our experiments can be explained by the heterogeneity of our primary cultures. In this regard, we identified cells that present pluripotent transcription factors OCT4, SOX2 and NANOG at early passages, both in the nuclei as well as in the cytoplasm, characteristics that were previously reported [7]. We found that as the number of passages increases, the transcription factor markers were confined specifically to the nucleus.

In our study, we evaluated only the percentage of cells that presented OCT4, SOX2 and NANOG in the nucleus, where the protein is functional [32]. The translocation from the nuclei to the cytoplasm and vice versa in pluripotent stem cells has been reported. OCT4, SOX2 and NANOG undergo post-translational modifications such as phosphorylation, sumolation, and ubiquitination [33–35]. For example, the phosphorylation of the transcription factor promotes its stability and favors its translocation into the nucleus [33, 34]. It will be very interesting to determine if these mechanisms are responsible for the heterogeneity of hAEC cultured *in vitro*. Furthermore, it has been proposed that heterogeneity is inherent to cells derived from the epiblast stage (mouse epiblast stem cells and hESC) [36, 37]. hAEC have been suggested to be derived from epiblast [2, 18, 19]; for this reason as well, hESC might possess an intrinsic memory of localization and proximity, and thus be primed to differentiate *in vitro* similar to the transient epiblast cells [37].

The pluripotent state is not only regulated by transcription factors; epigenetic mechanisms are also involved. Modifications of specific histone residues are recognized by transcription factors, thereby facilitating the binding of activator or repressor proteins to regions of chromatin that contain these marks. The methylation of H3 lysine 27 (K27) has been shown to be associated with gene silencing [38]. Antibody against H3K27me3 detects a closed state of the chromatin when cells lose their pluripotency and become committed to a specific differentiation phenotype [39, 40]; naïve hPSC have a nearly complete lack of this marker as compared with primed hPSC. The epigenetic state of amniotic epithelial cells was evaluated by immunocytochemistry with an anti-H3K27me3 and anti-H3K4me3 antibodies. Our results showed no significant differences among the passages. However, we observed a trend: the percentage of hAEC positive for H3K27me3 and H3K4me3 increased with later passages (Fig 5A and 5B). These results suggest that a subpopulation of cells are committed to a cell lineage, while other cells may maintain the pluripotency state and the possibility of bivalent promoters in hAEC.

### Differentiation of hAEC to progenitor of cortical neurons

We evaluated the differentiation to cortical progenitors with a two-stage protocol. We tested several combination of neural inductors to determine the percentage of Nestin-(neural stem cell marker) positive cells at the end of the proliferation stage in our cultures and found that only the condition with bFGF+Noggin+SB431542+EGF increased the number of positive cells as compared with the control. After 6 days without the growth factors or inhibitors, we identified cells committed to differentiate into cortical neurons, as indicated by their expression of the specific lineage markers TBR2, NeuN, β-III-tubulin and OTX2 (Fig 6). The differentiation of hAEC to neural linages was reported previously [3, 5, 7, 41, 42] based on studies that evaluated the number of cells positive for MAP-2, TUJ1, GFAP and Nestin by immunocytochemistry. To our knowledge the present study is the first to report the potential of hAEC to differentiate into progenitor of cortical neurons.

## Conclusions

Using a combination of cell surface markers, we identified a minority of the hAEC population with a capacity for self-renewal and the ability to express pluripotency-associated gene transcripts and proteins with ability to differentiate into progenitor of cortical neurons. These features are characteristic of what has been termed pluripotency. Considering these findings, the hAEC are a possible source of pluripotent stem cells that show characteristics similar to those of hESC: they constitute a heterogeneous population, they present proteins related to pluripotency as well as genes of naïve pluripotency and they can be differentiated to specific phenotypes (cortical neurons). This highlights the need to further investigate hAEC as a possible source of hPSC.

## Supporting Information

S1 Fig Morphology of hAEC and hESC cultured *in vitro* and expression of pluripotency markers in H1 line. (A) Microphotography of hAEC at P0. (B) Typical colony morphology of the H1 line. (C) H1 cells used as positive control for our immunocytochemistry antibodies against: OCT4, SOX2 and NANOG (red) as well as SSEA4, TRA-1-60 and E-cadherin (green). Nuclei were stained with DAPI (blue). Scale bar 50 μm.
(TIF)

S2 Fig Somatic cells not express genes associated with pluripotency. Representative images of the electrophoresis of RT-PCR products of mRNAs for transcription factors *OCT4* (151 bp), *SOX2* (264 bp), *NANOG* (286 bp), *REX1* (306 bp), *KLF4* (134 bp) and *GAPDH* (229 pb) of mouse embryonic fibroblast (MEF) and human peripheral blood (PB). L = ladder, H9 = hESC line H9 (positive control). As negative control (-RT), the reverse transcriptase enzyme was not added.
(TIF)

S1 Table Data of the RT-qPCR of the relative expression and standard error of the pluripotent transcription factors for H9 and hAEC cells at different passages. (DOCX)
