# Supplementary figures and images for: Markers of Pluripotency in Human Amniotic Epithelial Cells and Their Differentiation to Progenitor of Cortical Neurons
Source: PLoS One. 2015 Dec 31;10(12):e0146082. doi: 10.1371/journal.pone.0146082 (PMC4697857; doi:10.1371/journal.pone.0146082)

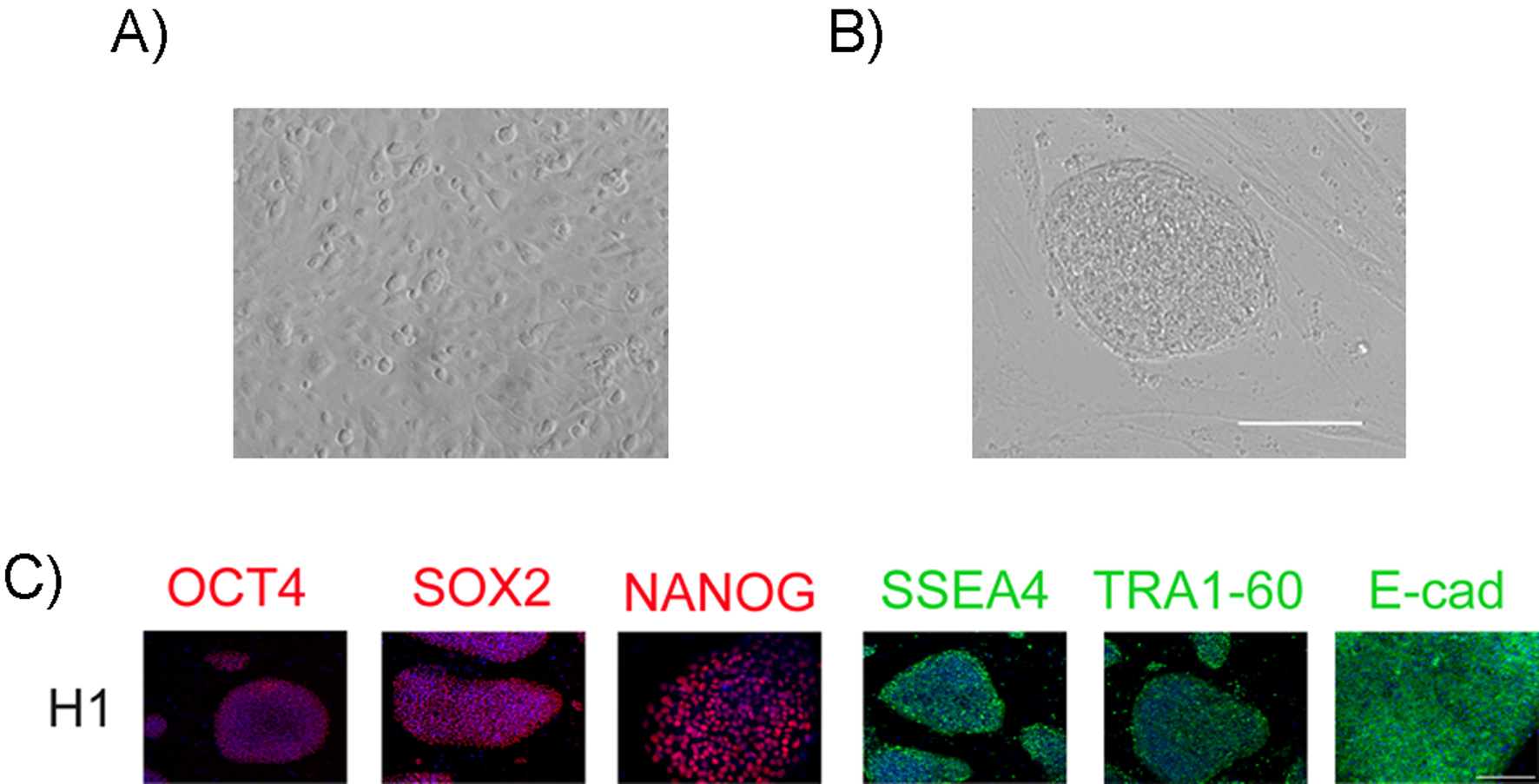

Supplement: S1 Fig — (A) Microphotography of hAEC at P0. (B) Typical colony morphology of the H1 line. (C) H1 cells used as positive control for our immunocytochemistry antibodies against: OCT4, SOX2 and NANOG (red) as well as SSEA4, TRA-1-60 and E-cadherin (green). Nuclei were stained with DAPI (blue). Scale bar 50 μm. (TIF) [file pone.0146082.s001.tif]

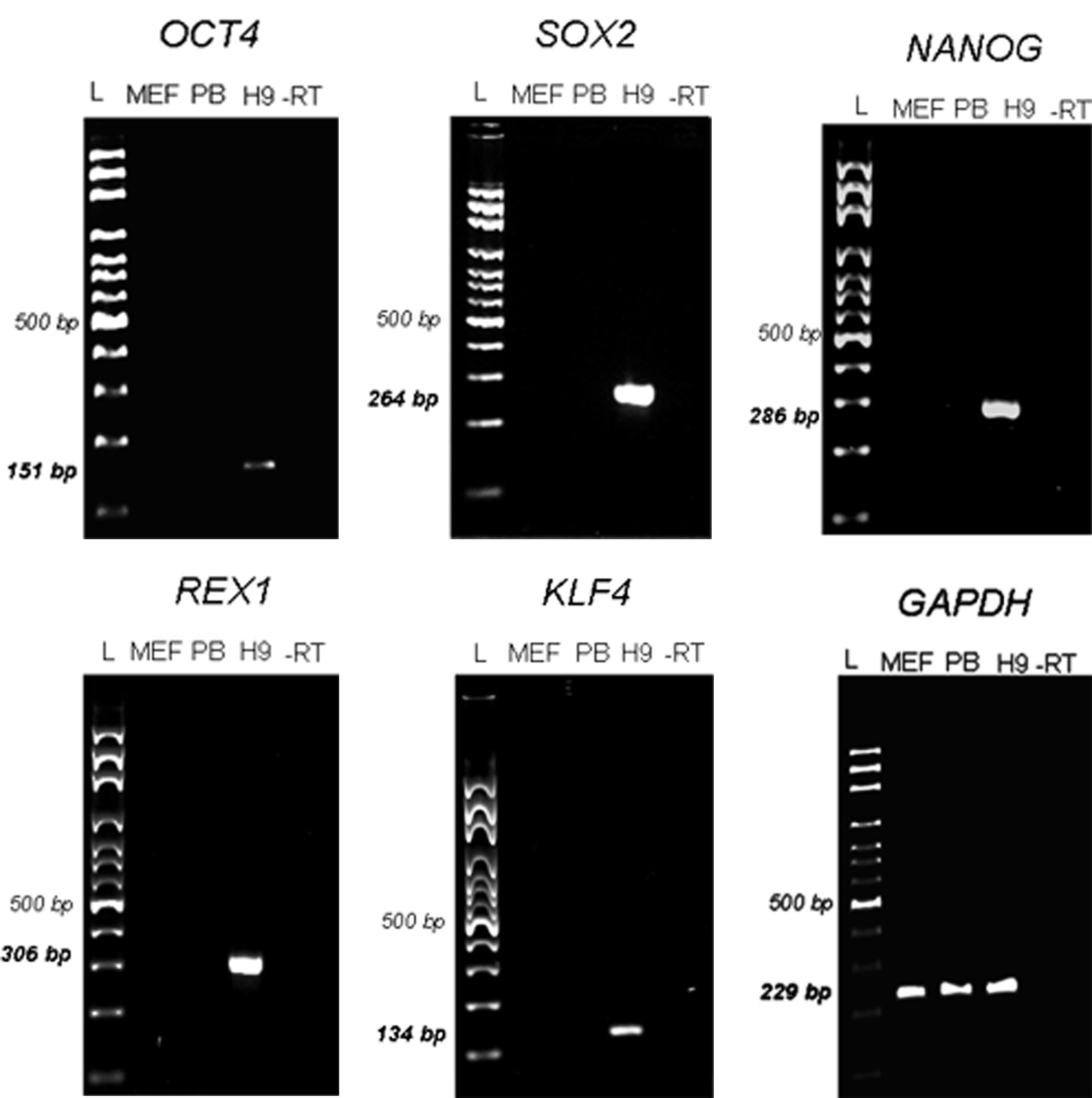

Supplement: S2 Fig — Representative images of the electrophoresis of RT-PCR products of mRNAs for transcription factors OCT4 (151 bp), SOX2 (264 bp), NANOG (286 bp), REX1 (306 bp), KLF4 (134 bp) and GAPDH (229 pb) of mouse embryonic fibroblast (MEF) and human peripheral blood (PB). L = ladder, H9 = hESC line H9 (positive control). As negative control (-RT), the reverse transcriptase enzyme was not added. (TIF) [file pone.0146082.s002.tif]
