# Supplementary material for: Markers of Pluripotency in Human Amniotic Epithelial Cells and Their Differentiation to Progenitor of Cortical Neurons
Source: PLoS One. 2015 Dec 31;10(12):e0146082. doi: 10.1371/journal.pone.0146082 (PMC4697857; doi:10.1371/journal.pone.0146082)
Supplement: S1 Table — (DOCX) [file pone.0146082.s003.docx]

|  | OCT4 | | SOX2 | | NANOG | | KLF4 | | REX1 | |
| --- | --- | --- | --- | --- | --- | --- | --- | --- | --- | --- |
|  | Relative expression | Standard error | Relative expression | Standard error | Relative expression | Standard error | Relative expression | Standard error | Relative expression | Standard error |
| H9 | 1.00 | ±0.157 | 1.00 | ±1.18 | 1.00 | ±0.076 | 1.00 | ±0.97 | 1.00 | ±1.7 |
| P0 | 0.86 | ±0.724 | 0.01 | ±1.99 | 0.12 | ±0.577 | 13.55 | ±5.03 | 1.22 | ±0.5 |
| P1 | 0.30 | ±0.617 | 0.00 | ±2.59 | 0.03 | ±0.97 | 3.59 | ±2.05 | 0.19 | ±0.59 |
| P2 | 0.19 | ±0.438 | 0.00 | ±1.34 | 0.07 | ±0.698 | 2.00 | ±7.64 | 0.21 | ±0.28 |
| P3 | 0.54 | ±1.386 | 0.02 | ±1.63 | 0.19 | ±0.45 | 2.54 | ±5.84 | 0.31 | ±8.76 |
